# Supplementary figures and images for: Essential Oil Content, Composition and Free Radical Scavenging Activity from Different Plant Parts of Wild Sea Fennel (Crithmum maritimum L.) in Montenegro
Source: Plants (Basel). 2024 Jul 22;13(14):2003. doi: 10.3390/plants13142003 (PMC11280542; doi:10.3390/plants13142003)

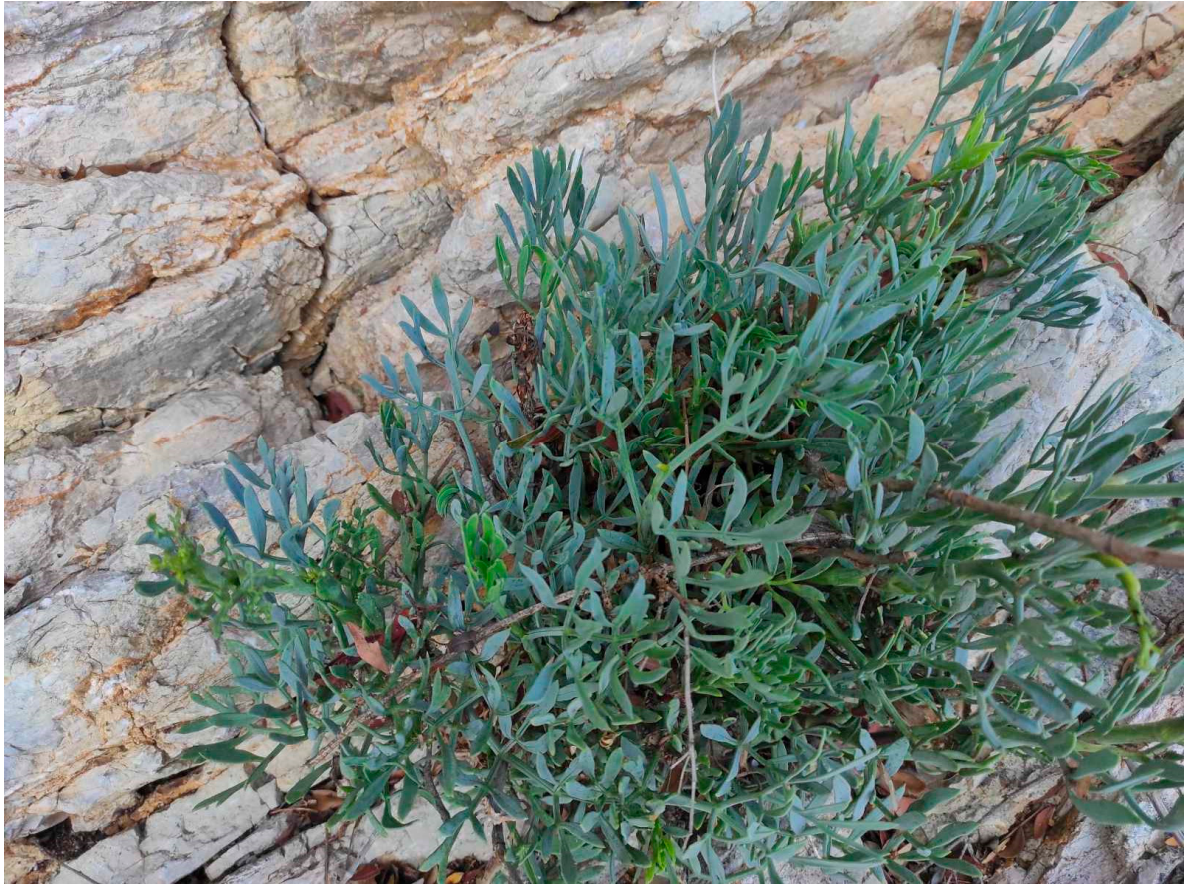

Figure S1. Sea fennel leaves

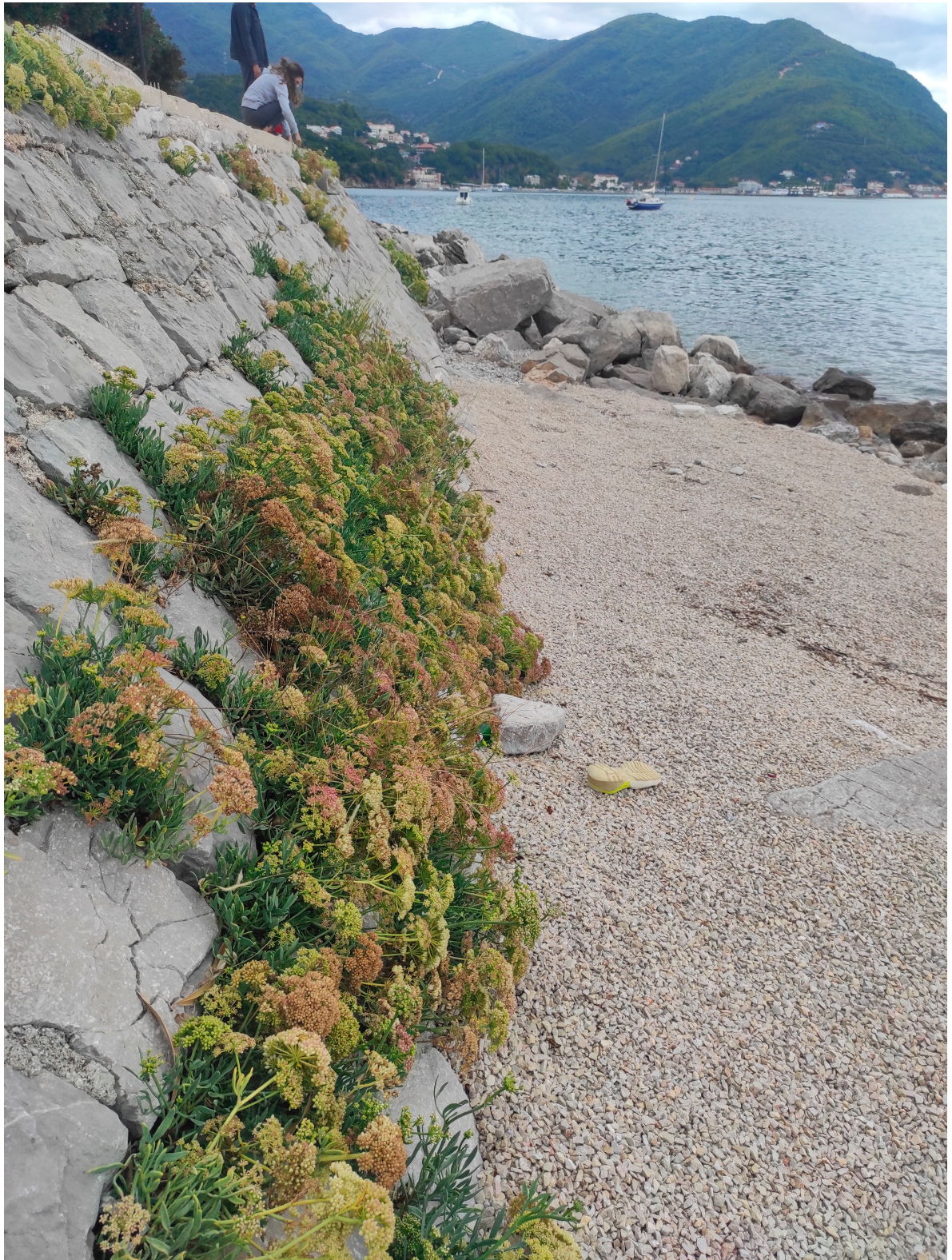

Figure S2. Sea fennel umbels with seed

Supplement: Supplementary file 1 [file plants-13-02003-s001.zip › plants-3087081-supplementary.pdf]
